# Long-Term Outcomes of Interventional Treatments for Lung Cancer Liver Metastases: Transarterial Chemoembolization and Microwave Ablation

**DOI:** 10.3390/jcm15197464

**Published:** 2026-09-25

**Authors:** Thomas J. Vogl, Angeliki M. Angelopoulou, Tatjana Gruber-Rouh, Catharina Recoschewitz, Hamzah Adwan

**Affiliations:** Clinic for Radiology and Nuclear Medicine, Goethe University, University Hospital Frankfurt, Theodor-Stern-Kai 7, 60590 Frankfurt am Main, Germany; angeliki.angelopoulou@unimedizin-ffm.de (A.M.A.); hamzah.adwan@unimedizin-ffm.de (H.A.)

**Keywords:** lung cancer, non-small cell carcinoma, small cell carcinoma, lung cancer liver metastases, transarterial chemoembolization, microwave ablation

## Abstract

**Purpose:** The aim of this study was to evaluate the long-term results of transarterial chemoembolization (TACE) and microwave ablation (MWA) as locoregional interventional treatments for lung cancer liver metastases (LCLM) in different protocols and settings. **Material and Methods:** A total of 56 patients (31 males and 25 females; mean age 59.1 ± 9.8 years) with LCLM were included in our retrospective study, conducted at our department between 2009 and 2024. These patients had undergone different therapies and were divided into two groups depending on the treatment protocol. Twenty-six patients (13 women and 13 men; mean age: 56.8 ± 11.7 years) with 49 tumors were solely treated by TACE, who underwent 77 sessions. A total of thirty patients (18 males and 12 females; mean age 61.2 ± 7.3 years) with 48 tumors were in the ablation-based group (TACE + MWA or MWA-only), who underwent 94 TACE and 53 MWA treatment sessions. **Results:** The ablation-based group exhibited significantly longer overall survival than the TACE-only group, achieving a median Overall Survival (mOS) of 1.6 years (95% CI: 1.1–2.1) versus 0.5 years (95% CI: 0.2–0.9) for the TACE-only group (Log-Rank χ^2^ = 12.17, *p* < 0.001). The survival rates at 1-, 3-, and 5-years were 70%, 23% and 8% for the ablation group, respectively, compared to 20%, 10%, and 0% for the TACE monotherapy group, in which no patient survived beyond 4.5 years. In a multivariable Cox model adjusted for histological subtype, number of lesions, and maximum lesion diameter, TACE monotherapy remained independently associated with mortality (hazard ratio [HR]: 2.711, 95% CI: 1.363–5.391, *p* = 0.004). With respect to neoadjuvant chemosensitivity, a per-lesion analysis revealed no statistically significant difference in tumor downsizing between Gemcitabine-based (12.6%) and Irinotecan-based (19.7%) regimens (*p* = 0.452). No major procedure-related complications or treatment-related deaths were identified in the available records across 224 treatment sessions. **Conclusions:** The ablation-based cohort exhibited significantly longer overall survival than the TACE monotherapy group, and the treatment group remained independently associated with survival after adjustment. Because treatment allocation followed hepatic tumor burden rather than randomization, this association is subject to confounding by indication and is interpreted accordingly. TACE was effective as a neoadjuvant downsizing modality with either chemotherapeutic combination, although the two were applied in different periods and were therefore not compared under equivalent conditions. These findings support the further evaluation of ablation-containing treatment strategies in appropriately selected patients and require confirmation in larger prospective studies.

## 1. Introduction

Lung cancer (LC) represents the second most frequently diagnosed cancer worldwide, after female breast cancer. In 2020, a total of over 2.2 million cases were identified, comprising 11.4% of the total number of diagnosed cancers, and 1.8 million deaths, accounting for 18.0% of the total number of cancer-related fatalities [1]. In patients with small cell lung cancer, the most common extrathoracic metastatic sites are bone (19–38%), followed by the liver, with an incidence of 17–34% [2]. According to the WHO classification, there are various types of lung tumors including non-small cell carcinoma (NSCLC) and small cell carcinoma (SCLC) [3].

Hepatic metastases have a poor prognostic value and usually have an undesirable response to systemic therapy. Therefore, liver resection in selected patients with lung cancer liver metastases (LCLM) has been reported to improve long-term survival [4]. However, only a minority of patients are suitable candidates to undergo resection [5].

In cases where surgical intervention is not feasible due to inoperable liver metastases, image-guided minimally invasive interventional procedures, such as microwave ablation (MWA) or transarterial chemoembolization (TACE), can be employed for local tumor treatment. In comparison to surgical resection, minimally invasive treatments have been shown to preserve healthy tissue, reduce costs, and be associated with fewer complications [6].

TACE is an interventional treatment in which chemoembolic materials are delivered through the arteries supplying the tumor. The result is selective ischemia and tumor necrosis [7]. In conventional TACE (cTACE), chemotherapeutic drugs are emulsified in lipiodol, followed by embolization to obstruct blood flow. Multiple sessions can be conducted to ensure complete tumor devascularization while sparing unaffected parts of the liver [8]. While numerous TACE techniques have evolved, cTACE and drug-eluting bead (DEB)-TACE remain the primary approaches [9]. DEB-TACE involves injecting DEBs into the hepatic artery of the mostly affected lobe, which act as embolic agents and gradually release chemotherapeutic drugs [8]. Although cTACE and DEB-TACE provide comparable outcomes [9], this study focuses on the effects of TACE, which can be performed as monotherapy or in combination with thermal ablation. 

One of the most common complications of TACE is Post-Embolization Syndrome, which includes symptoms such as fever, abdominal pain, nausea, and vomiting. This complication typically manifests after the procedure due to an inflammatory response to the embolization, and its duration can extend for several days [9]. It has been also observed that TACE may result in hepatic function deterioration, particularly in patients with underlying hepatic pathologies. The assessment of this condition is typically performed through the monitoring of liver enzymes and bilirubin levels. In rare instances, there is the potential for injury to the biliary tree, which can result in complications such as cholangitis or biliary obstruction. Other potential complications include hepatic arterial damage, which can result in hepatic artery thrombosis or systemic toxicity [9].

Nevertheless, careful patient selection and monitoring are imperative to mitigate these risks and ensure optimal outcomes for patients undergoing these procedures.

MWA is a hyperthermal treatment that uses an electromagnetic field radiating from the tip of an antenna. The high water content in the tissue causes microwaves to induce atomic and dipole rotation, creating friction between molecules. This molecular agitation raises the temperature, ultimately causing tumor cell death through coagulative necrosis in a specific ablation zone [10,11].

Complications associated with MWA for liver tumors have been documented and may result in significant morbidity, potentially requiring additional medical intervention or prolonged hospitalization [12]. Although most adverse events are minor and self-limiting, major complications have also been reported. These include thermal injury to adjacent tissues, biliary damage leading to bile leaks or strictures, hemorrhage, particularly when major blood vessels are involved, and infection [13]. Notably, in cases of ablation near the diaphragm, there is a risk of pneumothorax, defined as the presence of air in the pleural space. The mortality rate associated with MWA is low, ranging from 0% to 0.36% [14].

Overall, while MWA is considered a safe technique for the treatment of unresectable liver tumors, careful patient selection and procedural techniques are essential to minimize these complications.

This retrospective study aims to evaluate the long-term outcomes of different locoregional treatment strategies for liver metastases from lung cancer when applied according to their clinical and technical indications, and to identify the factors associated with overall survival in this population.

## 2. Materials and Methods

### 2.1. Ethical Statement

The current retrospective study was conducted in accordance with the Declaration of Helsinki and approved by the Ethics Committee of the Faculty of Medicine of the Goethe University Frankfurt (protocol code: 2025-2296 and date of approval: 19 February 2025).

### 2.2. Patients’ Selection Criteria

The inclusion criteria also encompassed the following: lung cancer as the sole primary tumor with liver metastasis confirmed by histological and/or radiological examination, the provision of informed consent, age over 18, non-response to systemic therapy, ability to undergo MRI on 1.5 or 3 Tesla scanners, initial tumor size greater than 1 cm, and clinically indicated treatment with TACE or MWA. Exclusion criteria comprised extrahepatic metastases or other known primary tumors, contraindications for MRI, pregnancy or breastfeeding status, renal insufficiency in stages 4 and 5, history of severe allergic reactions to contrast medium, and contraindications for TACE or MWA (e.g., poor health conditions, ascites, tumor involvement exceeding 70% of liver volume, advanced liver dysfunction, and complete portal vein thrombosis).

As a preliminary measure, the database underwent a filtration process to ensure the consistency, integrity, and completeness of the key selection criteria. The data elements deemed to be of particular significance included the dates of the first and last treatment, as well as the date of the patient’s death or last contact.

While these selection criteria constrained the final sample size, they facilitated a targeted analysis of this specific metastatic pattern. From the comprehensive dataset, patients were stratified into two distinct treatment groups for comparative analysis: the TACE-only group and the ablation-based group, where patients were treated with MWA either as monotherapy or in combination with TACE.

### 2.3. Treatment Selection Criteria

The choice of interventional strategy followed the technical indications for each modality. Thermal ablation was undertaken where the hepatic tumor burden permitted complete coagulative coverage of every target lesion with an adequate safety margin—in practice, five or fewer metastases with none exceeding 5 cm in maximum diameter at the time of ablation. Patients whose disease was within these limits at presentation underwent MWA as monotherapy.

Patients in the combination group (TACE + MWA) were presented with larger metastases, measuring more than 5 cm, and/or a higher hepatic tumor burden. These metastases were treated by ablation following successful downsizing through TACE. TACE was also used to reduce tumor vascularity and maximize the ablation effect in large lesions.

TACE monotherapy was applied where the criteria for ablation were not met at presentation, where adequate downsizing was not achieved, or where ablation was not undertaken for other clinical reasons.

### 2.4. TACE Technique

All patients were informed regarding the benefits, risks, side effects, and alternative therapeutic options, and informed consent was obtained.

The TACE procedure was initiated with the application of a local anesthetic after the inguinal region had been properly sterilized.

Following this, the femoral artery was punctured, and a catheter was carefully inserted through the artery using the Seldinger technique [8].

Diagnostic angiography of the abdominal aorta was initially performed to map the hepatic arterial supply [8].

Subsequently, a catheter was advanced through the hepatic artery to reach the tumor-feeding arteries directly, resulting in a higher intratumoral concentration of the chemotherapeutic agents [15].

In the cohort of 48 patients receiving TACE (either as monotherapy or in combination with MWA), the majority (n = 33) received a combination of Mitomycin C, Cisplatin, and Gemcitabine, while the remaining patients (n = 15) were treated with Mitomycin C, Cisplatin, and Irinotecan.

To achieve vascular occlusion and enhance chemotherapeutic retention [15], a maximum of 10 mL of Lipiodol was administered, followed by the injection of 30–225 mg of degradable starch microspheres (EmboCept^®^, PharmaCept GmbH, Berlin, Germany).

The choice of chemotherapeutic regimen was not randomized but reflected the evolution of institutional TACE practice over the study period [8,15]. Both protocols used Mitomycin C and Cisplatin as the backbone, combined with either Gemcitabine or Irinotecan. The two regimens were applied predominantly in different treatment periods: Gemcitabine-based TACE was used between 2009 and 2016, whereas Irinotecan-based TACE was used predominantly from 2017 onwards, with one patient receiving the Irinotecan-based regimen in 2012. The specific rationale for regimen selection at the individual-patient level was not systematically recorded in the study database and therefore cannot be reconstructed retrospectively.

Adequate vessel occlusion was confirmed by angiography. By the end of the TACE procedure, the inguinal region was closed using either a compression bandage or a percutaneous closure device.

The tumor response was a critical factor in deciding whether to pursue further interventions. Typically, three TACE sessions were performed as standard. If the tumor responded well to TACE or maintained a stable disease status, additional TACE procedures were conducted. However, if the tumor progressed after two consecutive TACE sessions, the interventions were discontinued [8].

A CT scan was performed within 24 h post-procedure to assess the distribution of the Lipiodol.

### 2.5. MWA Technique

Percutaneous MWA was performed by an experienced interventional radiologist under CT guidance (Siemens, Erlangen, Germany). Patients’ most recent abdominal contrast-enhanced CT and MRI scans as well as the latest hematologic and coagulation parameters were reviewed before ablation. To ensure the best approach for the MWA antenna, an unenhanced abdominal CT scan was acquired immediately prior to the intervention [16].

After the injection of local anesthesia, the microwave antenna was percutaneously inserted into the target LCLM lesion through a small skin incision under sterile conditions. After the accurate positioning of the antenna, thermal ablation was performed [16]. In accordance with the established protocol, the ablation procedure was initiated with a low energy setting and subsequently augmented to a higher power level [17]. The vital signs were continuously monitored during the procedure.

Subsequent to the completion of the ablation session, track ablation was performed during the withdrawal of the antenna to induce coagulation and minimize the risk of tumor seeding along the needle path. In the absence of complications that necessitated continuous observation or therapeutic interventions, patients were discharged on the same day of treatment [16]. Treatment efficacy was evaluated via contrast-enhanced MRI 24 h post-procedure. In instances where incomplete ablation resulted in residual tumor, a subsequent MWA session was scheduled.

### 2.6. Statistical Methods

The collection of data and management of the resulting database were performed using Microsoft Excel. Statistical analyses were conducted using IBM SPSS Statistics (Version 26.0) and RStudio (R version 4.4.1) using the packages survival (version 3.8-12) and survminer (version 0.5.2) [18,19]. Propensity-score matching was performed using MatchIt (version 4.7.2), with covariate balance assessed using cobalt (version 5.0.0); E-values were calculated using the EValue package (version 4.1.4). Descriptive statistics were used to summarize the data. Continuous variables are presented as mean ± standard deviation (SD) or median with interquartile range (IQR), as appropriate, whereas categorical variables are expressed as frequencies and percentages. All statistical tests were two-sided, and a *p*-value ≤ 0.05 was considered statistically significant.

The Kaplan–Meier survival estimator was employed to analyze overall survival [20]. The overall survival time was calculated from the date of the initial interventional treatment session to the date of death or last follow-up. Patients who were lost to follow-up or were still alive at the conclusion of the study period were censored at the date of their last contact. The survival curves between the treatment groups (TACE-only vs. ablation-based) were compared using the Log-Rank test [21]. An exploratory Kaplan–Meier analysis was also performed for TACE-only, MWA-only, and TACE + MWA, with an overall log-rank test. The proportional-hazards assumption was assessed using scaled Schoenfeld residuals.

To identify prognostic factors and quantify the risk of mortality, univariate and multivariate Cox proportional hazards regression models were employed [22]. The results of this study are reported as hazard ratios (HR) with 95% confidence intervals (CI).

Regarding the evaluation of tumor response, the percentage of tumor downsizing was calculated for each target lesion (n = 94). A comparison of the efficacy of downsizing between two different chemotherapeutic regimens (Gemcitabine-based vs. Irinotecan-based) was conducted using the Mann–Whitney U test.

Treatment allocation was determined by clinical and technical indications described in Section 2.3 rather than by randomization. Consequently, between-group comparisons are therefore interpreted as associations rather than as estimates of relative treatment efficacy. The primary analysis compared the TACE-only group with the ablation-based cohort, which comprised the MWA-only and TACE + MWA subgroups. The MWA-only subgroup, which included only eight patients with four events, was deemed too imprecise to support reliable standalone inference. It was therefore retained within the ablation-based cohort for the main grouped analysis, while its separate survival curve and summary estimates were reported descriptively. An exploratory three-group Kaplan–Meier analysis of TACE-only, MWA-only, and TACE + MWA was additionally performed with an overall log-rank test; no formal pairwise comparisons were performed. As a restricted sensitivity analysis, the multivariable Cox model was repeated after excluding the MWA-only subgroup and comparing TACE + MWA with TACE-only, to assess whether the association observed in the grouped analysis persisted.

Effect sizes for the between-regimen comparison are reported as the rank-biserial correlation and as the Hodges–Lehmann estimate of the median difference with its 95% confidence interval, computed in R. Because no exclusion rule had been specified in advance, the comparison was repeated with all measured lesions as a sensitivity analysis.

Sensitivity analyses included propensity-score matching for allocation to TACE monotherapy. Propensity scores were estimated using logistic regression including lesion number, maximum lesion diameter, histological subtype, and age. One-to-one nearest-neighbor matching without replacement was performed using MatchIt [23], with a caliper width of 0.2 standard deviations of the logit of the propensity score [24]. Covariate balance before and after matching was assessed using standardized mean differences (SMDs), with an absolute SMD < 0.10 considered indicative of adequate balance. The treatment association in the matched sample was estimated using a Cox proportional hazards model with matching weights and robust standard errors clustered by matched pair.

Additional sensitivity analyses included a model adjusted for calendar year of first treatment. Robustness to unmeasured confounding was quantified using the E-value [25].

## 3. Results

### 3.1. Treatments

A total of 56 patients (31 males and 25 females; mean age 59.1 ± 9.8 years) with LCLM were treated between 2009 and 2024 in our department. Patients’ characteristics, including age and sex and number of treatments received, are presented for the three treatment modalities in Table 1.

A total number of 26 patients (46.4%) were treated only with TACE in 77 sessions (average 3.0 TACE sessions per patient). The group consisted of 13 women and 13 men, with a mean age of 56.8 ± 11.7 years.

The ablation-based group comprised 30 patients (53.6%), including those treated with MWA monotherapy (n = 8) and those receiving a combination of TACE + MWA (n = 22). This group consisted of 12 women and 18 men, ranging from 40 to 73 years, with a mean age of 61.2 ± 7.3 years. Specifically, the MWA monotherapy subgroup underwent 10 sessions, while the combination subgroup received 94 TACE and 43 MWA sessions. Overall, patients in the ablation-based group were slightly older compared to the TACE-only group. With respect to the tumor characteristics, the most commonly treated type of primary lung metastatic malignancy was NSCLC, as indicated by 41 patients (73.2%). SCLC was observed in 15 patients (26.8%).

The total number of tumors treated was 97. The TACE-only group targeted 49 tumors (50.5%), while the ablation-based group targeted 48 tumors (49.5%). The TACE group presented the largest tumor burden, demonstrating a mean diameter of 4.6 ± 3.2 cm, considerably larger than the ablation-based group (mean 3.0 ± 1.9 cm).

Lesion size and technical suitability for complete ablation, rather than lesion count, therefore, appear to have been the determinants of treatment allocation. The two groups did not differ materially in the number of hepatic lesions (mean 1.88 versus 1.60), whereas the mean tumor diameter differed substantially (4.6 ± 3.2 cm versus 3.0 ± 1.9 cm).

In the present study, patients who received MWA as monotherapy (subgroup of ablation-based) had predominantly one lesion (7/8 patients). The size of these lesions did not exceed 3.0 cm (mean 2.0 ± 0.6 cm) at the time of the initial presentation. These patients underwent curative intent therapy, and the final assessment revealed complete response (CR) in 7 out of the 8 patients. This subgroup had substantially lower hepatic tumor burden than patients treated with TACE or combination therapy.

Conversely, TACE monotherapy was applied to patients with less favorable hepatic disease characteristics, either because the criteria for ablation were not met at presentation or because downsizing did not bring the disease within the ablation criteria. In the present study, in our cohort (N = 26), six patients (23.1%) had a single lesion, 17 (65.4%) had two, and three (11.5%) had three. No patient in this group presented with more than three hepatic lesions.

Lesions were larger on average, with a mean diameter of 4.6 cm. Consequently, according to RECIST 1.1 evaluation (Table 2), none of the patients in the TACE cohort achieved complete response (0%), with the majority showing stable disease (12/26, 46.2%) or progressive disease (11/26, 42.3%).

In contrast, in the ablation-based group (MWA and TACE + MWA combined), complete response was reported in 21 out of 30 patients (70.0%), with 5 patients (16.7%) exhibiting progressive disease (Table 2).

### 3.2. Chemotherapeutics

To assess the specific cytoreductive efficacy of the chemotherapeutic regimens, a per-lesion analysis was conducted on the cohort. One lesion, with a maximum diameter of 14.72 cm, lay far outside the distribution of the remaining lesions and was not included in the primary comparison. Because no exclusion rule had been defined in advance, the comparison was repeated with this lesion included as a sensitivity analysis. The lesion was retained in all survival and prognostic analyses.

The final comparative analysis included a total of 94 target lesions. Group 1 comprised 71 lesions treated with the Gemcitabine-based regimen (Mitomycin C, Cisplatin, and Gemcitabine). Group 2 comprised 23 lesions treated with the Irinotecan-based regimen, which consisted of Mitomycin C, Cisplatin, and Irinotecan.

The individual response of all 94 target lesions is detailed in the waterfall plot in Figure 1.

A descriptive analysis revealed a mean tumor size reduction of 12.6% (SD ± 39.6) for the Gemcitabine group and 19.7% (SD ± 30.9) for the Irinotecan group. Notwithstanding the numerical discrepancy, the Mann–Whitney U test revealed no statistically significant discrepancy in downsizing efficacy between the two groups (U = 731.000, Z = −0.752, *p* = 0.452) [26].

The Hodges–Lehmann estimate of the median difference between the regimens was 5.5 percentage points in favor of the Irinotecan-based combination (95% CI −10.6 to +22.6), and the rank-biserial correlation was 0.11. When the analysis was repeated with the excluded lesion restored (95 lesions; 72 Gemcitabine-based and 23 Irinotecan-based), the mean reductions were 12.5% and 19.7%, the Mann–Whitney U was 738 (Z = −0.782, *p* = 0.434), the median difference was 5.6 percentage points (95% CI −10.3 to +22.8), and the rank-biserial correlation was 0.11. The result was unchanged whether the lesion was included or not.

No significant difference in downsizing was detected between the two regimens (*p* = 0.452). This comparison should not be interpreted as a causal comparison of drug efficacy. Because the two combinations were used in different periods (Section 2.4), the contrast between them is confounded with calendar time and therefore with the interventional technology, imaging resolution, and systemic treatment options available in each era. Given this, the non-randomized allocation, and the modest number of lesions treated with the Irinotecan-based regimen, the results represent an absence of evidence for a difference rather than evidence of equivalence. The confidence interval on the median difference is compatible with the Irinotecan-based regimen being associated with approximately 10 percentage points less to 23 percentage points greater tumor downsizing.

### 3.3. Survival Analysis

Overall survival by treatment protocol is shown in Figure 2, and the corresponding estimates are presented in Table 3.

As illustrated in Table 3, the study presents the overall survival time in years as mean and median values, as well as the 1-, 3-, and 5-year survival rates (in percent) with 95% confidence intervals according to the specific treatment modality.

The resultant analysis yielded a *p*-value of less than 0.001, which is significantly less than the 5% threshold. These findings demonstrate statistical significance, indicating a substantial discrepancy in the survival distributions favoring the ablation-based protocol.

TACE-only group: This cohort, comprising 26 patients and 20 events of death (76.9%), exhibited a median overall survival time of 0.5 years (mean overall survival time = 0.9 years) over the course of the study. The 1- and 3-year survival probabilities were 20% (95% CI: 6–40%) and 10% (95% CI: 2–27%), respectively. No patient survived the 5-year mark (Table 3).

Separated into the three treatment modalities (Figure 2B), 4 out of the 8 patients in the MWA-monotherapy subgroup, 15 out of 22 in the TACE + MWA group, and 20 out of 26 in the TACE-monotherapy group died during follow-up. Median overall survival was 2.5, 1.3, and 0.5 years, respectively; the corresponding 1-year survival probabilities were 83%, 65%, and 20%, and the 3-year probabilities 42%, 20%, and 10%. The overall log-rank test across the three groups was significant (χ^2^ = 13.08, 2 df, *p* = 0.001). These estimates are presented descriptively; the MWA-monotherapy subgroup comprised eight patients and its curve was not used for formal comparison.

The ablation-based group: The consolidated ablation group (MWA and TACE + MWA), comprising 30 patients and 19 events of death (63.3%) over the study period, exhibited a significantly prolonged median overall survival time of 1.6 years (mean overall survival time = 2.1 years). The estimated 1-, 3-, and 5-year survival probabilities were 70% (95% CI: 48–84%), 23% (95% CI: 7–44%), and 8% (95% CI: 1–28%), respectively.

A multivariate Cox proportional hazards regression analysis was conducted to evaluate the impact of treatment modality, primary lung tumor histology, and hepatic tumor burden with survival (Table 4).

Treatment group remained independently associated with survival: patients treated by TACE monotherapy had a hazard ratio of 2.711 (95% CI 1.363–5.391, *p* = 0.004) relative to the ablation-based group. Histological subtype and maximum lesion diameter were also independently associated with survival, whereas the number of hepatic lesions was not. Given the 39 observed events, the primary multivariable model was restricted to four clinically relevant covariates available for all patients: treatment group, histological subtype, maximum lesion diameter, and number of hepatic lesions. Age and sex were examined separately but were not included in the primary model to avoid overfitting.

In a restricted sensitivity analysis excluding the MWA-monotherapy subgroup, patients treated with TACE + MWA (n = 22) were compared with patients treated with TACE monotherapy (n = 26). The median overall survival was 1.3 versus 0.5 years (log-rank χ^2^ = 7.80, *p* = 0.005). The hazard ratio for TACE monotherapy was 2.620 (95% CI 1.304–5.266, *p* = 0.007) unadjusted and 2.567 (95% CI 1.244–5.297, *p* = 0.011) after adjustment for histological subtype, number of lesions, and maximum lesion diameter (Table 4). The association observed in the grouped analysis was also present after exclusion of the MWA-only subgroup.

Table 5 and Table 6 present the unadjusted associations of the treatment methods, patient and tumor characteristics, and tumor histology as univariate models. Specifically, Table 5 shows that TACE monotherapy and maximum lesion diameter were each associated with survival, whereas the number of hepatic lesions, age, and sex were not. Table 6 reports the association with histological subtype without adjustment for other covariates.

In the univariate analysis of treatment modalities (Table 5), the TACE-only approach was associated with a hazard ratio of 3.030 (95% CI: 1.582–5.803, *p* < 0.001). This indicates a threefold increase in the risk of mortality compared to the ablation-based group. Treatment group was significantly associated with survival in the univariate model.

Regarding the primary tumor’s histology (Table 6), the univariate model identified the type of lung cancer as a statistically significant prognostic factor: with NSCLC as the reference category, patients with SCLC had a hazard ratio of 2.014 (95% CI 1.030–3.936, *p* = 0.041). The association remained significant in the multivariable model (HR 2.085, *p* = 0.035) after adjustment for treatment group and tumor burden (Table 4).

### 3.4. Safety

Across the cohort, 224 liver-directed treatment sessions were performed, comprising 171 TACE sessions and 53 MWA procedures. A dedicated complication field was available for 128 treatment occasions across all 56 patients; no complication was recorded on 126 occasions. Two minor complications occurred in one patient treated by TACE monotherapy and in one patient treated by TACE followed by MWA. Neither event required additional treatment or prolonged hospitalization, neither resulted in permanent sequelae, and neither met the criteria for a major complication as defined in the standardized reporting criteria for image-guided tumor ablation [12]. No major complication or treatment-related death was identified in the available records.

Adverse events were recorded descriptively in the procedural and imaging reports rather than captured prospectively against a defined instrument. The complication field was not available for every session, and post-embolization syndrome was not documented as a discrete variable. The rate of minor events may therefore be underestimated, and these data are reported as evidence of the absence of major complications rather than as a systematic safety analysis.

### 3.5. Sensitivity Analyses

Because treatment allocation was governed by hepatic tumor burden rather than by randomization, the comparison between groups is subject to confounding by indication. A propensity-score analysis was performed to examine how far this accounts for the observed association.

Propensity scores for allocation to TACE monotherapy were estimated by logistic regression including the number of hepatic lesions, maximum lesion diameter, histological subtype, and age. Baseline imbalance was present across all four covariates, with absolute standardized mean differences (SMDs) of 0.484, 0.516, 0.307, and 0.379, respectively, each exceeding the conventional threshold of 0.10.

The propensity-score distributions overlapped only partially. Forty-four out of 56 patients fell within the region of common support, including 18 out of 30 patients in the ablation-based group and all 26 patients in the TACE-only group. Three patients in the TACE-only group had a maximum lesion diameter of 8.1, 11.0, and 14.7 cm, exceeding the largest baseline lesion treated in the ablation-based group (8.0 cm before downsizing by TACE), illustrating the limited availability of comparable patients at the upper end of tumor burden. One-to-one nearest-neighbor matching without replacement using a caliper of 0.2 standard deviations of the logit of the propensity score retained 15 matched pairs (30 patients), leaving 26 patients unmatched. After matching, balance improved substantially, although residual imbalance remained for lesion number (|SMD| = 0.111) and age (|SMD| = 0.217); maximum lesion diameter (|SMD| = 0.044) and histology (|SMD| = 0.000) were adequately balanced. The matched sample included 23 events. In the matched Cox model, TACE monotherapy was associated with an HR of 1.642 (95% CI 0.759–3.551; *p* = 0.208). The confidence interval included the null and remained wide, indicating substantial uncertainty in the matched estimate.

The limited propensity-score overlap, the reduced matched sample, and the residual imbalance in lesion number and age indicate that propensity-score matching reduced but did not eliminate confounding by indication.

Two further checks were therefore performed. Because patients receiving TACE monotherapy were treated earlier than those undergoing ablation (median year of first treatment 2012 versus 2016.5), the primary model was additionally adjusted for calendar year; the association with treatment group persisted (HR 2.731, 95% CI 1.258–5.927, *p* = 0.011) while calendar year itself was not significant (*p* = 0.97). Finally, an E-value was calculated to quantify robustness to the confounding that no adjustment can remove: an unmeasured confounder would need to be associated with both treatment allocation and mortality at a risk ratio of at least 3.37, over and above the measured covariates, to account for the observed association in full (Table 7).

## 4. Discussion

LC is the leading cause of cancer-related deaths worldwide [1]. Liver metastases are associated with poor prognosis and may adversely affect outcomes with immune checkpoint inhibitors in advanced lung cancer [27].

The management of liver metastases exhibits significant variability, encompassing a range of therapeutic modalities such as liver transplantation, surgical resection, chemotherapy, immunotherapy, radiotherapy, and ablation [28].

To the best of our knowledge, this is the first retrospective single-center study with long-term follow-up evaluating TACE monotherapy, MWA monotherapy, and combined TACE + MWA in patients with LCLM. The survival observed in the ablation-based cohort provides additional long-term outcome data in the context of previously reported local therapies.

Zhang et al. (2022) investigated the clinical value of percutaneous radiofrequency ablation for LCLM, reporting a median overall survival of 1.1 years with 1-, 3- and 5-year survival rates of 55%, 22%, and 14% [29]. The median overall survival of 1.6 years in our ablation-based group lies within the range reported for thermal ablation in this indication. The two cohorts differ in eligibility criteria, in the period over which they were treated, and in the systemic options available to them, and the difference between them should not be read as a comparison of ablation modalities.

The association between treatment pathway and survival was also observed when the analysis was restricted to patients treated with TACE + MWA and those treated with TACE alone (adjusted HR 2.567, 95% CI 1.244–5.297, *p* = 0.011). This restricted analysis indicates that the main grouped association was also present after exclusion of the small MWA-only subgroup. However, because entry into the combination pathway depended on response to TACE and survival to subsequent ablation, this analysis cannot establish a causal benefit of adding MWA.

This is consistent with results reported from our institution for the combination of transarterial embolization and microwave ablation in hepatocellular carcinoma, where combination therapy achieved significantly longer overall survival than ablation alone [30].

Patients diagnosed with NSCLC and liver metastases typically have a poor prognosis [28,31], and the outcomes reported for systemic therapy in this population provide the clinical context in which locoregional treatment is considered.

Ileana et al. (2010) highlighted the uncertainty surrounding hepatic resection for NSCLC liver metastases, while suggesting that selected patients may achieve long-term survival after resection [32]. Systemic immunotherapy provides an important clinical context for patients with NSCLC and liver metastases. A review of previously treated patients reported median overall survival ranging from 3.6 to 7.5 months across studies of nivolumab in patients with liver metastases [33]. Zhu et al. (2024) summarized a separate cohort of 1096 patients with NSCLC and liver metastases, in which the 6-month survival rate was 68.2% [34]. In a real-world study of 648 patients with advanced NSCLC treated with PD-1/PD-L1 inhibitors, patients with liver metastases had a median overall survival of 15.2 months compared with 20.6 months in patients without liver metastases [35]. These findings are reported as clinical context rather than a direct comparison with the present cohort. The populations differed substantially in treatment setting, hepatic tumor burden, performance status, extrahepatic disease, and systemic treatment. Therefore, no inference regarding the relative efficacy of locoregional versus systemic therapy can be drawn from these cross-study observations. 

With respect to histological subtypes, liver metastasis has been identified as an independent prognostic factor across all groups. As reported by Fan et al. (2024), patients with SCLC and liver metastases exhibit a dismal prognosis, with a median overall survival of merely three months [36].

Xia et al. (2022) observed that while liver metastases do not negate the benefits of immunotherapy in NSCLC, they may limit efficacy in SCLC [27]. In our multivariate analysis, both histological subtype and treatment group remained associated with survival. Because the model does not include performance status, comorbidity, or concurrent systemic therapy, and because treatment allocation followed hepatic tumor burden, this association is hypothesis-generating rather than an estimate of a treatment effect.

Several mechanisms could be considered, although they remain speculative in the present study. At the local level, thermal ablation is designed to achieve complete coagulative destruction of technically suitable target lesions with an adequate safety margin, whereas TACE primarily achieves arterial devascularization and cytoreduction. A possible systemic mechanism has also been proposed: experimental studies suggest that liver metastases may suppress antitumor immunity through the macrophage-mediated elimination of activated CD8+ T cells, and that liver-directed treatment may modulate this effect [37]. However, the present study includes no immunological correlates and cannot determine whether either mechanism contributed to the observed survival association. Residual differences in disease biology and patient selection remain alternative explanations.

The IMpower150 trial, which proved to be crucial in this regard, demonstrated that the combination of atezolizumab, bevacizumab, and chemotherapy led to an improvement in median overall survival for patients with liver metastases, with a notable increase to 13.2 months [38].

Hu et al. (2024) proposed a Phase 2 trial protocol combining ultrasound-guided High-Intensity Focused Ultrasound (HIFU) with PD-1 inhibitors to enhance outcomes in patients with liver metastases from lung cancer [39].

In a similar study, Yin et al. (2022) reported median overall survival ranges of 12.6–13.3 months for ICI combinations [40].

Regarding the optimization of the interventional protocol, we further investigated whether the choice of chemotherapeutic agent influenced the degree of tumor downsizing—a critical factor for minimizing the heat-sink effect during subsequent ablation. No statistically significant difference was detected between the Gemcitabine- and Irinotecan-based protocols (*p* = 0.452). Because the regimens were not randomized and were applied predominantly in different treatment periods, and because the confidence interval on the median difference is wide, this observation is descriptive rather than a demonstration of equivalence and does not permit conclusions about the relative contribution of the specific cytotoxic agent to the overall effect of TACE.

Liao et al. (2025) reported a median overall survival of 19.6 months in patients treated with thermal ablation combined with immune checkpoint inhibitors [41]. This study illustrates the current interest in combining liver-directed and systemic therapies; however, its population and treatment setting are not directly comparable with the present cohort.

These observations require prospective validation. Future studies should assess combined TACE and MWA in cohorts stratified by lesion diameter, histological subtype and hepatic tumor burden, and should be sized to separate the contribution of each modality—a question the present cohort, in which allocation was determined by tumor burden, cannot address.

## 5. Limitations

This study was subject to several limitations inherent to its retrospective single-center design.

First, and most importantly, treatment allocation was not independent of prognosis. Ablation was offered where lesion diameter and geometry permitted complete thermal coverage, and TACE monotherapy was reserved for patients in whom this was not achievable. The comparison between the two groups is therefore confounded by indication, and the observed hazard ratio cannot be interpreted as the causal effect of ablation. Propensity-score analysis does not resolve this, because the covariates governing allocation are the same variables that determine prognosis, leaving limited common support between the groups.

Assignment to the combination group was, in addition, determined in part by events occurring after the start of treatment: a patient entered that group only if the hepatic disease responded to transarterial treatment sufficiently for ablation to become technically feasible, and only if the patient survived long enough for the ablation to be undertaken. Seven out of the 26 patients treated by TACE monotherapy died within three months of their first treatment. The combination group is therefore enriched, by construction, for patients whose disease responded and whose course permitted a second intervention. This introduces a form of guarantee-time bias, because patients classified to the TACE + MWA pathway had to survive and remain eligible until the subsequent ablation. This is a structural feature of the treatment pathway rather than a deficiency of measurement; it cannot be removed by adjustment for baseline covariates, and it has been described previously for interventional cohorts treated under comparable selection criteria at our institution [42]. The comparison should accordingly be read as a comparison of treatment pathways as delivered under their clinical and technical indications, and not as an estimate of the effect of adding ablation to transarterial treatment in an unselected patient.

Second, the sample size was small, particularly within the MWA-monotherapy subgroup (n = 8), which limits statistical power and generalizability. Conclusions drawn from cohorts of this size should be interpreted with caution until validated in larger studies [43].

Third, performance status, comorbidity burden, and concurrent systemic therapy are not recorded in the study database and could not be included as covariates. These are established prognostic factors in metastatic lung cancer, and part of the observed survival difference is likely attributable to them. The E-value reported in Section 3.5 quantifies how strong such an unmeasured confounder would need to be to account for the association in full. The E-value addresses potential unmeasured confounding but does not account for this structural selection or guarantee-time bias.

Fourth, the study spanned 15 years, during which ablation devices, microwave generators and imaging resolution advanced, and the systemic treatment options available to patients with metastatic lung cancer changed substantially. This temporal heterogeneity was not evenly distributed across the cohort: patients treated with TACE monotherapy were treated earlier than those who underwent ablation. The two chemotherapeutic regimens were used predominantly in different treatment periods. Calendar year was associated with survival in univariate analysis (HR 0.899, 95% CI 0.819–0.987). In a sensitivity model additionally adjusted for year of first treatment, the association between treatment group and survival persisted (HR 2.731, 95% CI 1.258–5.927), and calendar year was no longer significant; this model is reported as a sensitivity analysis rather than as the primary model because five covariates exceed what 39 events reliably support.

Fifth, although the involved liver segment was recorded for every patient, proximity of the target lesion to major hepatic vasculature was not systematically documented. An analysis of the heat-sink effect on local recurrence therefore remains beyond the scope of these data.

Sixth, progression-free survival could not be estimated reliably, for two reasons. Imaging follow-up intervals were determined clinically rather than by protocol and varied considerably between patients and across the study period, so any PFS estimate would carry interval-censoring bias of unknown magnitude and direction. In addition, after microwave ablation, the coagulation zone and its reactive rim were measured as lesion diameter, so an increase in measured size did not distinguish residual or recurrent tumor from expected post-ablation change. Local response was therefore summarized using RECIST 1.1 at the available post-procedural imaging assessments (Table 2), rather than as a time-to-progression endpoint.

Finally, adverse-event capture was retrospective and not based on a predefined instrument, so the data in Section 3.4 support the absence of major complications in the available records but do not constitute a systematic safety analysis.

## 6. Conclusions

This long-term single-center analysis found that ablation-based treatment pathways were associated with longer overall survival than TACE monotherapy in patients with liver metastases from lung cancer. Treatment group remained associated with survival after adjustment for histological subtype and hepatic tumor burden, and an association of similar magnitude was observed when the analysis was restricted to patients treated with TACE + MWA versus TACE alone. Small cell histology and larger maximum lesion diameter were also associated with shorter survival. Tumor downsizing was observed with both TACE chemotherapeutic combinations, although the regimens were used predominantly in different treatment periods and were not compared under equivalent conditions. No major procedure-related complications were identified in the available records, although adverse-event capture was retrospective and incomplete. These findings must be interpreted in the context of the treatment-selection process described in Section 2.3: TACE monotherapy was used when ablation criteria were not met or adequate downsizing was not achieved, and entry into the TACE + MWA pathway additionally depended on response to TACE and survival to subsequent ablation. The observed survival associations therefore should not be interpreted as causal treatment effects. Prospective multicenter studies with predefined treatment pathways, standardized follow-up, and more complete clinical and systemic-treatment data are required to establish the role of combined transarterial and ablative treatment in this setting.

## Figures and Tables

**Figure 1:**
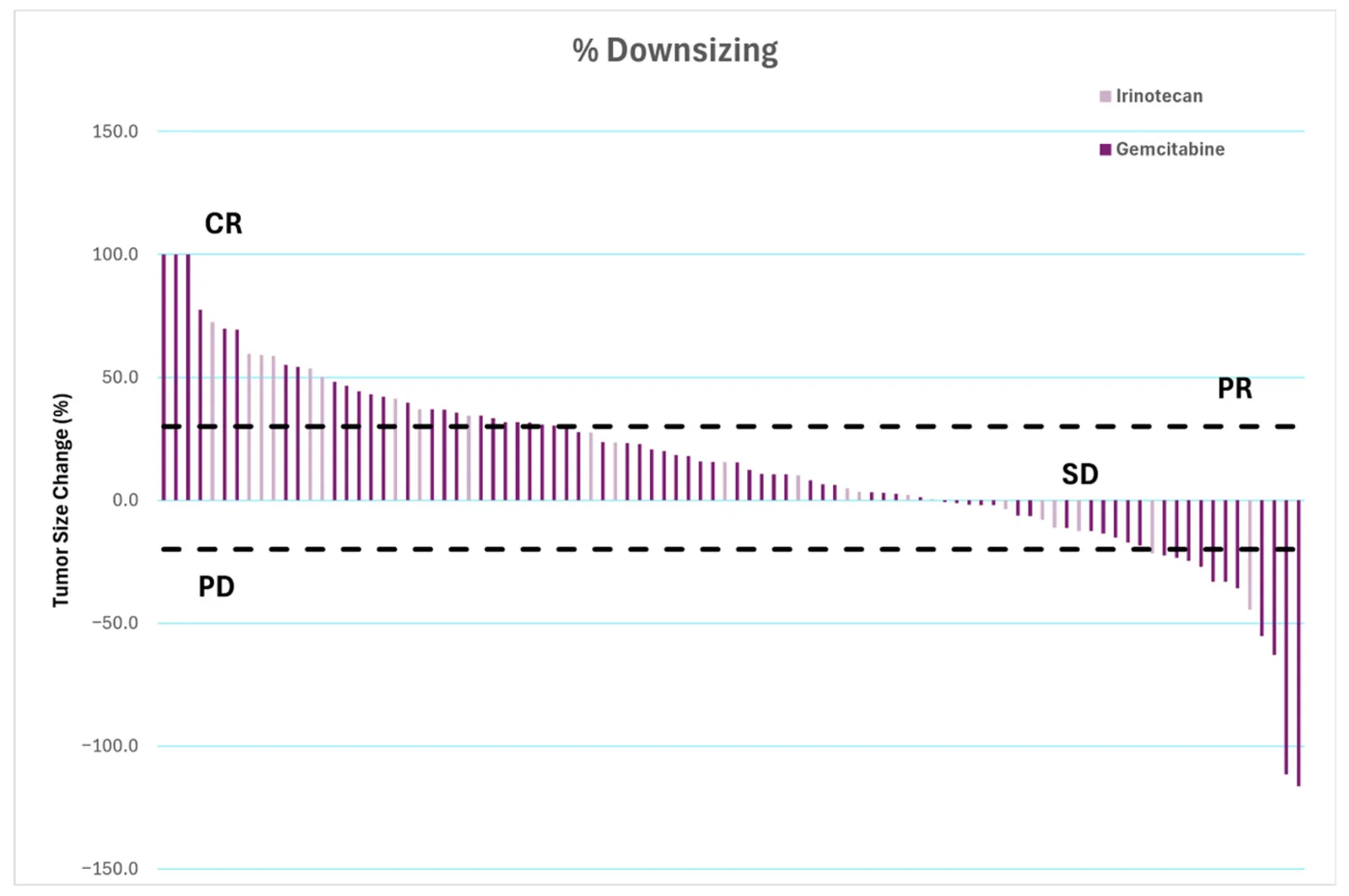
The waterfall plot illustrates the optimal tumor response. The waterfall plot shows the percentage change in diameter of all analyzed target lesions (n = 94) following TACE treatment. The vertical axis of this graph indicates the percentage change from the baseline. The dashed lines represent the thresholds for partial response and progressive disease, as defined by the RECIST 1.1 criteria. Lesions that occur between these lines are classified as stable disease (SD). CR denotes complete response. The bars are color-coded according to the specific chemotherapeutic agent: dark purple indicates Gemcitabine, and light purple signifies Irinotecan. Most lesions exhibited disease control (CR + PR + SD) following TACE.

**Figure 2:**
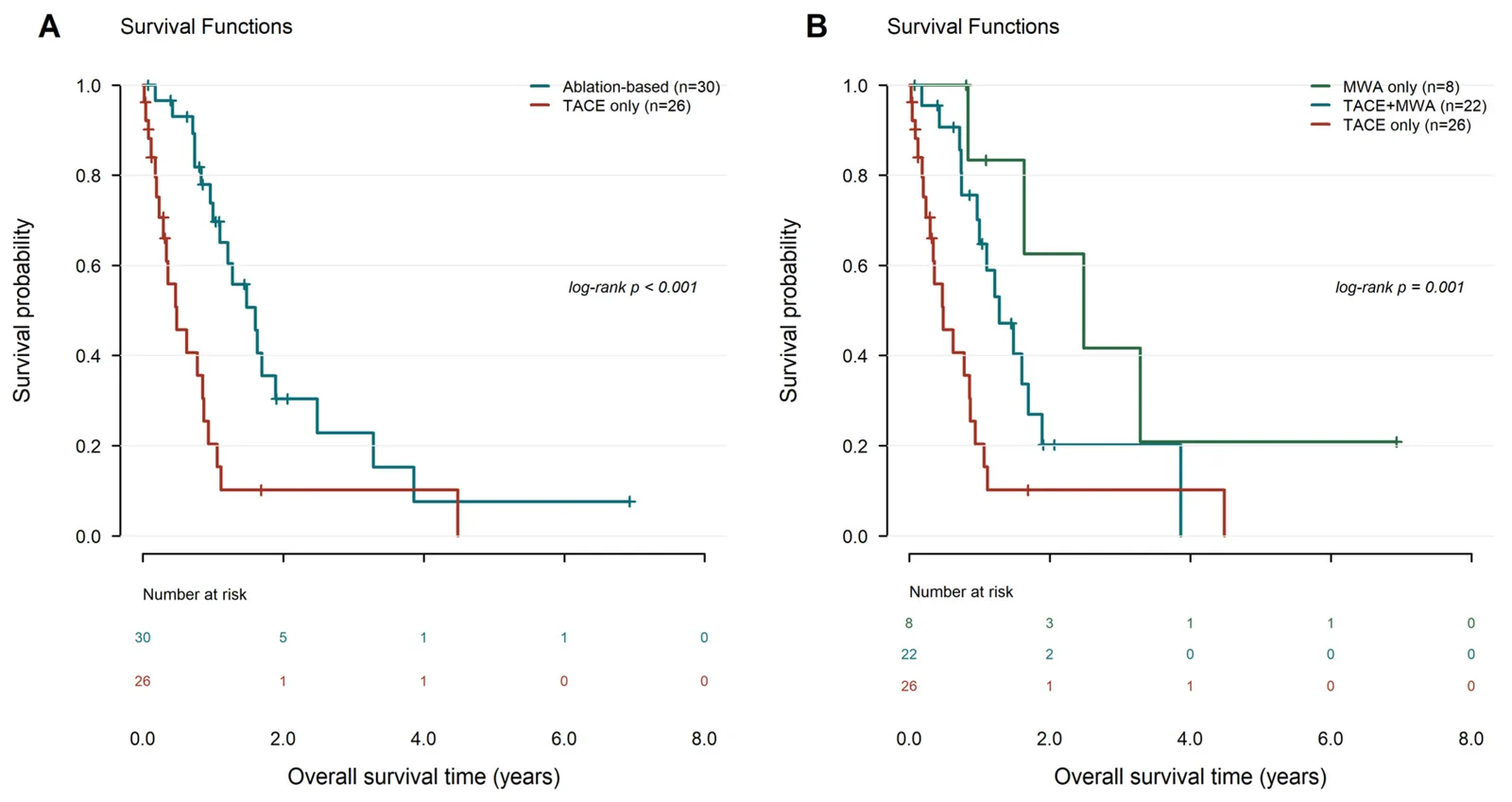
Kaplan–Meier estimates of overall survival. (**A**) Primary comparison between the ablation-based and TACE-only protocols. (**B**) The same cohort separated into the three treatment modalities. Vertical marks denote censored observations. Time is calculated from the first interventional session. The MWA-monotherapy subgroup comprised eight patients, seven of whom presented with a single lesion of ≤3 cm; its curve is shown for descriptive completeness, and the confidence intervals around it are too wide to support inference.

**Table 1:** Characteristics of the patients.

Variables	TACE	MWA	TACE + MWA	Ablation Based	Total
**Patients’ data, n (%)**	26 (46.4)	8 (14.3)	22 (39.3)	30 (53.6)	56 (100)
**Age (years),** mean ± SD	56.8 ± 11.7	59.0 ± 9.2	62.0 ± 6.6	61.2 ± 7.3	59.1 ± 9.8
**Sex, n (%)**					
**Male**	13 (50.0)	4 (50.0)	14 (63.6)	18 (60.0)	31 (55.4)
**Female**	13 (50.0)	4 (50.0)	8 (36.4)	12 (40.0)	25 (44.6)
**Type of primary lung metastatic malignancy, n (patients, %)**					
**NSCLC**	17 (65.4)	7 (87.5)	17 (77.3)	24 (80.0)	41 (73.2)
**SCLC**	9 (34.6)	1 (12.5)	5 (22.7)	6 (20.0)	15 (26.8)
**No. of tumors treated (tumors, %)**	49 (50.5)	9 (9.3)	39 (40.2)	48 (49.5)	97 (100.0)
**Lesions per patient**					
**1, n (%)**	6 (23.1)	7 (87.5)	13 (59.1)	20 (66.7)	26 (46.4)
**2, n (%)**	17 (65.4)	1 (12.5)	3 (13.6)	4 (13.3)	21 (37.5)
**3, n (%)**	3 (11.5)	0	5 (22.7)	5 (16.7)	8 (14.3)
**5, n (%)**	0	0	1 (4.5)	1 (3.3)	1 (1.8)
**Mean diameter of the tumors**					
**diameter (cm),** mean ± SD	4.6 ± 3.2	2.0 ± 0.6	3.3 ± 2.0	3.0 ± 1.9	3.7 ± 2.6
**TACE chemotherapeutic drug combined with Mitomycin C, Cisplatin**					
**Gemcitabine (patients, %)**	22 (84.6)		11 (50.0)	11 (50.0)	33 (68.8)
**Irinotecan (patients, %)**	4 (15.4)		11 (50.0)	11 (50.0)	15 (31.2)
**No. of TACE cycles**	77		94	94	171
**Average no. of TACE sessions per TACE-treated patient**	3		4.3	4.3	3.6
**No. of MWA cycles**		10	43	53	53
**Average no. of MWA treatments per patient**		1.3	2.0	1.8	1.8

n: number; TACE: transarterial chemoembolization; MWA: microwave ablation; Combined: TACE + MWA; NSCLC: non-small cell lung carcinoma; SCLC: small cell lung carcinoma. MWA and TACE + MWA are subgroups of the ablation-based cohort; the ablation-based column is their aggregate and is not an additional patient group. Cohort totals therefore reflect the two mutually exclusive groups, TACE-only and ablation-based. Session averages are calculated among patients receiving the respective modality; chemotherapy percentages among TACE-treated patients only (n = 48).

**Table 2:** Final assessment of tumor response in compliance with RECIST 1.1.

Final Assessment-RECIST 1.1					
Therapy Protocol	TACE	MWA	TACE + MWA	Ablation-Based	Total n (%)
Complete response	0	7	14	21	21 (37.5)
Partial response	3	0	1	1	4 (7.1)
Stable disease	12	0	3	3	15 (26.8)
Progressive disease	11	1	4	5	16 (28.6)
Total	26	8	22	30	56 (100.0)

The ABLATION-BASED column is the sum of the MWA and TACE + MWA columns and does not represent an additional group of patients; the TACE and ABLATION-BASED columns together comprise the whole cohort. Complete response was recorded in no patient treated by TACE alone and in 21 out of 30 patients in the ablation-based group. This difference is in part definitional, since thermal ablation aims at complete coagulative necrosis of the target lesion whereas transarterial chemoembolization achieves cytoreduction; the response categories of the two strategies are therefore not directly comparable.

**Table 3:** Mean values, median, and 1-/3-/5-year survival rates (%) with 95% confidence interval by treatment method. Data for the number of events (death of the patients).

Variable	TACE Only	Ablation-Based	Total
**Number of patients**	26	30	56
**Number of events (n, %)**	20 (76.9)	19 (63.3)	39 (69.6)
**Mean overall survival time (years)**	0.9	2.1	1.6
**Median overall survival time (years)**	0.5	1.6	1.0
**Std. error, SE (years)**	0.2	0.2	0.1
**Median, 95% lower CI**	0.2	1.1	0.7
**Median, 95% upper CI**	0.9	2.1	1.3
**1-year survival rate (%)**	20	70	
**1-year 95% lower CI**	6	48	
**1-year 95% upper CI**	40	84	
**3-year survival rate (%)**	10	**23**	
**3-year 95% lower CI**	2	7	
**3-year 95% upper CI**	27	44	
**5-year survival rate (%)**	**0**	**8**	
**5-year 95% lower CI**		1	
**5-year 95% upper CI**		28	

n: number; TACE-only: only transarterial chemoembolization; ABLATION-BASED: microwave ablation and transarterial chemoembolization; CI: confidence interval; SE: standard error. Survival estimated by the Kaplan–Meier method; confidence intervals by the log–log transformation. Log-rank χ^2^ = 12.17, df = 1, *p* < 0.001. Mean overall survival is the restricted mean, calculated to the longest observed follow-up time in each group. No patient in the TACE-only group was followed beyond 4.5 years, at which point all had died; the 5-year estimate for that group is therefore 0 by observation.

**Table 4:** Multivariable Cox regression analyses.

Predictor	Hazard Ratio	Lower 95% CI	Upper 95%CI	*p*-Value
Primary model (n = 56, 39 events)				
Therapy (TACE only vs. ablation-based)	2.711	1.363	5.391	0.004
Histology (SCLC vs. NSCLC)	2.085	1.053	4.126	0.035
Maximum lesion diameter, per 1 cm	1.213	1.043	1.411	0.012
Number of hepatic lesions, per lesion	1.274	0.848	1.914	0.243
Restricted sensitivity model: TACE + MWA vs. TACE monotherapy (n = 48, 35 events)				
Therapy (TACE only vs. TACE + MWA)	2.567	1.244	5.297	0.011
Histology (SCLC vs. NSCLC)	2.299	1.119	4.723	0.024
Maximum lesion diameter, per 1 cm	1.215	1.035	1.425	0.017
Number of hepatic lesions, per lesion	1.281	0.848	1.934	0.240

Primary model: Events per variable = 9.75. Model concordance = 0.74 (SE 0.038); likelihood ratio test χ^2^ = 20.84 on 4 df, *p* < 0.001. The proportional hazards assumption was examined using scaled Schoenfeld residuals and was not violated (global χ^2^ = 4.95, 4 df, *p* = 0.29; no individual covariate *p* < 0.08). Restricted sensitivity model: Patients treated by MWA monotherapy (n = 8) were not included in this model.

**Table 5:** Univariate Cox regression analysis, treatment and patient characteristics.

Predictors	*p*-Value	Hazard Ratio	Lower 95% CI	Upper 95% CI
Therapy group	<0.001	3.030	1.582	5.803
Maximum lesion diameter, per 1 cm	0.009	1.215	1.050	1.406
Number of hepatic lesions, per lesion	0.157	1.246	0.919	1.691
Age, per year	0.354	0.985	0.953	1.017
Sex (male vs. female)	0.342	0.728	0.379	1.401

**Table 6:** Univariate Cox regression analysis regarding primary lung tumor histology (unadjusted).

Predictors	*p*-Value	Hazard Ratio	Lower 95% CI	Upper 95% CI
Histology (SCLC vs. NSCLC)	0.041	2.014	1.030	3.936

NSCLC is the reference category. Small cell lung cancer (SCLC).

**Table 7:** Sensitivity analyses for the treatment-group comparison.

Analysis	HR, TACE Only	95% CI	*p*
Primary model (Table 4)	2.711	1.363–5.391	0.004
Additionally adjusted for year of first treatment	2.731	1.258–5.927	0.011
Propensity-score matched, 15 pairs	1.642	0.759–3.551	0.208

Propensity scores estimated from lesion number, maximum diameter, histology, and age. E-value for the primary estimate = 3.37; 1.78 for the confidence limit closest to the null.

## Data Availability

The data presented in this study are available on request from the corresponding author. The data are not publicly available due to privacy and ethical restrictions.

## References

[1] Sung H., Ferlay J., Siegel R.L., Laversanne M., Soerjomataram I., Jemal A., Bray F. (2021). Global Cancer Statistics 2020: GLOBOCAN Estimates of Incidence and Mortality Worldwide for 36 Cancers in 185 Countries. CA Cancer J. Clin..

[2] Tas F., Aydiner A., Topuz E., Camlica H., Saip P., Eralp Y. (1999). Factors influencing the distribution of metastases and survival in extensive disease small cell lung cancer. Acta Oncol..

[3] Nicholson A.G., Tsao M.S., Beasley M.B., Borczuk A.C., Brambilla E., Cooper W.A., Dacic S., Jain D., Kerr K.M., Lantuejoul S. (2022). The 2021 WHO Classification of Lung Tumors: Impact of Advances Since 2015. J. Thorac. Oncol..

[4] Ercolani G., Ravaioli M., Grazi G.L., Cescon M., Varotti G., Del Gaudio M., Vetrone G., Zanello M., Principe A., Pinna A.D. (2006). The role of liver resections for metastases from lung carcinoma. HPB.

[5] Adam R., Vinet E. (2004). Regional treatment of metastasis: Surgery of colorectal liver metastases. Ann. Oncol..

[6] Bale R., Putzer D., Schullian P. (2019). Local Treatment of Breast Cancer Liver Metastasis. Cancers.

[7] Cho Y., Choi J.W., Kwon H., Kim K.Y., Lee B.C., Chu H.H., Lee D.H., Lee H.A., Kim G.M., Oh J.S. (2023). Transarterial chemoembolization for hepatocellular carcinoma: 2023 expert consensus-based practical recommendations of the Korean Liver Cancer Association. J. Liver Cancer.

[8] Vogl T.J., Adwan H., Wolff L., Lahrsow M., Gruber-Rouh T., Nour-Eldin N.-E.A., Trojan J., Bechstein W.-O., Naguib N.N.N. (2024). Retrospective Long-Term Evaluation of Conventional Transarterial Chemoembolization for Hepatocellular Carcinoma over 20 Years. Cancers.

[9] Song J.E., Kim D.Y. (2017). Conventional vs drug-eluting beads transarterial chemoembolization for hepatocellular carcinoma. World J. Hepatol..

[10] Gölz O., Radler P., Deininger J., Lebhardt P., Langejürgen J. (2024). Development of an intraoperative monitoring system for microwave ablations. Curr. Dir. Biomed. Eng..

[11] Simon C.J., Dupuy D.E., Mayo-Smith W.W. (2005). Microwave ablation: Principles and applications. Radiographics.

[12] Goldberg S.N., Grassi C.J., Cardella J.F., Charboneau J.W., Dodd G.D., Dupuy D.E., Gervais D.A., Gillams A.R., Kane R.A., Lee F.T. (2009). Image-guided Tumor Ablation: Standardization of Terminology and Reporting Criteria. J. Vasc. Interv. Radiol..

[13] Fang C., Cortis K., Yusuf G.T., Gregory S., Lewis D., Kane P., Peddu P. (2019). Complications from percutaneous microwave ablation of liver tumours: A pictorial review. Br. J. Radiol..

[14] Izzo F., Granata V., Grassi R., Fusco R., Palaia R., Delrio P., Carrafiello G., Azoulay D., Petrillo A., Curley S.A. (2019). Radiofrequency Ablation and Microwave Ablation in Liver Tumors: An Update. Oncologist.

[15] Vogl T.J., Lahrsow M. (2022). The Role of Conventional TACE (cTACE) and DEBIRI-TACE in Colorectal Cancer Liver Metastases. Cancers.

[16] Adwan H., Hammann L., Vogl T.J. (2023). Microwave Ablation of Recurrent Hepatocellular Carcinoma after Curative Surgical Resection. J. Clin. Med..

[17] Vogl T.J., Nour-Eldin N.-E.A., Hammerstingl R.M., Panahi B., Naguib N.N.N. (2017). Microwave Ablation (MWA): Basics, Technique and Results in Primary and Metastatic Liver Neoplasms—Review Article. Rofo.

[18] Therneau T.M. (2024). survival: Survival Analysis.

[19] Kassambara A., Kosinski M., Biecek P. (2025). survminer: Drawing Survival Curves Using ‘ggplot2’.

[20] Chakraborty S. (2018). A step-wise guide to performing survival analysis. Cancer Res. Stat. Treat..

[21] Lin R.S., León L.F. (2017). Estimation of treatment effects in weighted log-rank tests. Contemp. Clin. Trials Commun..

[22] Emmert-Streib F., Dehmer M. (2019). Introduction to Survival Analysis in Practice. Mach. Learn. Knowl. Extr..

[23] Ho D.E., Imai K., King G., Stuart E.A. (2011). MatchIt: Nonparametric Preprocessing for Parametric Causal Inference. J. Stat. Softw..

[24] Austin P.C. (2011). Optimal caliper widths for propensity-score matching when estimating differences in means and differences in proportions in observational studies. Pharm. Stat..

[25] VanderWeele T.J., Ding P. (2017). Sensitivity Analysis in Observational Research: Introducing the E-Value. Ann. Intern. Med..

[26] Krauth J. (1983). The interpretation of significance tests for independent and dependent samples. J. Neurosci. Methods.

[27] Xia H., Zhang W., Zhang Y., Shang X., Liu Y., Wang X. (2022). Liver metastases and the efficacy of immune checkpoint inhibitors in advanced lung cancer: A systematic review and meta-analysis. Front. Oncol..

[28] Tsilimigras D.I., Brodt P., Clavien P.A., Muschel R.J., D’Angelica M.I., Endo I., Parks R.W., Doyle M., de Santibañes E., Pawlik T.M. (2021). Liver metastases. Nat. Rev. Dis. Primers.

[29] Zhang Z.-Y., Jiang A.-N., Yang W., Yan K., Wu W., Wang S., Jiang B.-B., Sun L.-Q., Zhao K., Chen M.-H. (2022). Percutaneous Radiofrequency Ablation Is an Effective Method for Local Control of Liver Metastases from Lung Cancer. Front. Oncol..

[30] Adwan H., Adwan M., Vogl T.J. (2023). Combination Therapy of Bland Transarterial Embolization and Microwave Ablation for Hepatocellular Carcinoma within the Milan Criteria Leads to Significantly Higher Overall Survival. Cancers.

[31] Liao Y., Fan X., Wang X. (2019). Effects of different metastasis patterns, surgery and other factors on the prognosis of patients with stage IV non-small cell lung cancer: A Surveillance, Epidemiology, and End Results (SEER) linked database analysis. Oncol. Lett..

[32] Ileana E., Greillier L., Moutardier V., Barlesi F. (2010). Surgical resection of liver non-small cell lung cancer metastasis: A dual weapon?. Lung Cancer.

[33] Ćeriman Krstić V., Samardžić N., Gajić M., Savić M., Šeha B., Roksandić Milenković M., Jovanović D. (2024). Treatment Options for Patients with Non-Small Cell Lung Cancer and Liver Metastases. Curr. Issues Mol. Biol..

[34] Zhu L., Yu X., Tang X., Hu C., Wu L., Liu Y., Zhou Q. (2024). Evolving landscape of treatments targeting the microenvironment of liver metastases in non-small cell lung cancer. Chin. Med. J..

[35] Xie M., Li N., Xu X., Xu Y., Li H., Zhu L., Sheng J., Zhou Z., Fan Y. (2022). The Efficacy of PD-1/PD-L1 Inhibitors in Patients with Liver Metastasis of Non-Small Cell Lung Cancer: A Real-World Study. Cancers.

[36] Fan L., Lin Y., Fu Y., Wang J. (2024). Small cell lung cancer with liver metastases: From underlying mechanisms to treatment strategies. Cancer Metastasis Rev..

[37] Yu J., Green M.D., Li S., Sun Y., Journey S.N., Choi J.E., Rizvi S.M., Qin A., Waninger J.J., Lang X. (2021). Liver metastasis restrains immunotherapy efficacy via macrophage-mediated T cell elimination. Nat. Med..

[38] Nogami N., Barlesi F., Socinski M.A., Reck M., Thomas C.A., Cappuzzo F., Mok T.S., Finley G., Aerts J.G., Orlandi F. (2022). IMpower150 Final Exploratory Analyses for Atezolizumab Plus Bevacizumab and Chemotherapy in Key NSCLC Patient Subgroups with EGFR Mutations or Metastases in the Liver or Brain. J. Thorac. Oncol..

[39] Hu C., Fu Q., Gao F.F., Zeng J., Xiao W., Li H., Peng L., Huang X., Yang L., Chen W.Z. (2024). Ultrasound-Guided High-Intensity Focused Ultrasound Combined with PD-1 Blockade in Patients with Liver Metastases from Lung Cancer: Protocol for a Single-Arm Phase 2 Trial. JMIR Res. Protoc..

[40] Yin Q., Dai L., Sun R., Ke P., Liu L., Jiang B. (2022). Clinical Efficacy of Immune Checkpoint Inhibitors in Non–Small Cell Lung Cancer Patients with Liver Metastases: A Network Meta-Analysis of Nine Randomized Controlled Trials. Cancer Res. Treat..

[41] Liao Y., Wang D., Yang X., Ni L., Lin B., Zhang Y., Feng G., Li J., Gao F., Liao M. (2025). High-intensity focused ultrasound thermal ablation boosts the efficacy of immune checkpoint inhibitors in advanced cancers with liver metastases: A single-center retrospective cohort study. Oncol. Lett..

[42] Vogl T.J., Freichel J., Gruber-Rouh T., Nour-Eldin N.-E.A., Becker S., Solbach C., Stefenelli U., Naguib N.N.N. (2023). Interventional oncological treatment of breast cancer liver metastasis (BCLM): Single center long-term evaluation over 26 years using thermoablation techniques like LITT, MWA and TACE in a multimodal application. Int. J. Hyperth..

[43] Hackshaw A. (2008). Small studies: Strengths and limitations. Eur. Respir. J..

